# Supplementary material for: Deletion of Von Hippel–Lindau Interferes with Hyper Osmolality Induced Gene Expression and Induces an Unfavorable Gene Expression Pattern
Source: Cancers (Basel). 2020 Feb 12;12(2):420. doi: 10.3390/cancers12020420 (PMC7073186; doi:10.3390/cancers12020420)
Supplement: Supplementary file 1 [file cancers-12-00420-s001.zip › cancers-675002-supplementary revised/cancers-675002-supplementary revised.docx]

**Supplemental Materials**

**Table S1.** Sequences selected for cloning and amplification of targeted region.

| gRNA oligos | Sequence (5´-3´) | Targeted Exon |
| --- | --- | --- |
| *Vhl*-gRNA1-Oligo1 | CAC CGA CAA AGG CAG CAC GAC GCG C | 1 |
| *Vhl*-gRNA1-Oligo2 | AAA CGC GCG TCG TGC TGC CTT TGT C |  |
| *Vhl*-gRNA2-Oligo1 | CAC CGA CCG AGC GCA GCA CCG GCC G | 1 |
| *Vhl*-gRNA2-Oligo2 | AAA CCG GCC GGT GCT GCG CTC GGT C |  |
| *Vhl*-gRNA3-Oligo1 | CAC CGT AGG CTG TCC ATC GAC ATT G | 2 |
| *Vhl*-gRNA3-Oligo2 | AAA CCA ATG TCG ATG GAC AGC CTA C |  |
| *Scrambled*-gRNA-Oligo1 | CAC CGA TAT CCG GAA TTC GCG CGA T | - |
| *Scrambled*-gRNA-Oligo2 | AAA CAT CGC GCG AAT TCC GGA TAT C |  |
| PCR oligos |  |  |
| *Vhl* gRNA1-2-PCR-S | CGA GGG ACC CGT TCC AAT AA |  |
| *Vhl* gRNA1-2-PCR-AS | ATG CAG TGG GTA GGG ACA AG |  |
| *Vhl* gRNA3-PCR-S | GAC CCG TGT GGC TTC TTA CT |  |
| *Vhl* gRNA3-PCR-AS | CTC ACC CCT CTT TTT CAA AGG C |  |

Methods

The next generation sequencing and data analysis was performed by Novogene. This was performed as provided and described by Novogene.

RNA Quantification and Qualification

RNA degradation and contamination was monitored on 1% agarose gels. RNA purity was checked using the NanoPhotometer® spectrophotometer (IMPLEN, CA, USA). RNA integrity and quantitation were assessed using the RNA Nano 6000 Assay Kit of the Bioanalyzer 2100 system (Agilent Technologies, CA, USA).

Library Preparation for Transcriptome Sequencing

A total amount of 1 μg RNA per sample was used as input material for the RNA sample preparations. Sequencing libraries were generated using NEBNext® UltraTM RNA Library Prep Kit for Illumina® (New England Biolabs, Ipswich, MA, USA) following manufacturer’s recommendations and index codes were added to attribute sequences to each sample. Briefly, mRNA was purified from total RNA using poly-T oligo-attached magnetic beads. Fragmentation was carried out using divalent cations under elevated temperature in NEBNext First Strand Synthesis Reaction Buffer (5X). First strand cDNA was synthesized using random hexamer primer and M-MuLV Reverse Transcriptase (RNase H-). Second strand cDNA synthesis was subsequently performed using DNA Polymerase I and RNase H. Remaining overhangs were converted into blunt ends via exonuclease/polymerase activities. After adenylation of 3’ ends of DNA fragments, NEBNext Adaptor with hairpin loop structure were ligated to prepare for hybridization. In order to select cDNA fragments of preferentially 150~200 bp in length, the library fragments were purified with AMPure XP system (Beckman Coulter, Beverly, USA). Then 3 μl USER Enzyme (New England Biolabs, USA) was used with size-selected, adaptor ligated cDNA at 37 °C for 15 min followed by 5 min at 95 °C before PCR. Then PCR was performed with Phusion High-Fidelity DNA polymerase, Universal PCR primers and Index (X) Primer. At last, PCR products were purified (AMPure XP system) and library quality was assessed on the Agilent Bioanalyzer 2100 system.

Clustering and Sequencing

The clustering of the index-coded samples was performed on a cBot Cluster Generation System using PE Cluster Kit cBot-HS (Illumina, San Diego, CA, USA) according to the manufacturer’s instructions. After cluster generation, the library preparations were sequenced on an Illumina platform and paired-end reads were generated.

Data Analysis and Quality Control

Raw data (raw reads) of FASTQ format were firstly processed through in-house scripts. In this step, clean data (clean reads) were obtained by removing reads containing adapter and poly-N sequences and reads with low quality from raw data. At the same time, Q20, Q30 and GC content of the clean data were calculated. All the downstream analyses were based on the clean data with high quality.

Mapping to Reference Genome

Reference genome and gene model annotation files were downloaded from genome website browser (NCBI/UCSC/Ensembl) directly. Paired-end clean reads were mapped to the reference genome using HISAT2 software. HISAT2 uses a large set of small GFM indexes that collectively cover the whole genome. These small indexes (called local indexes), combined with several alignment strategies, enable rapid and accurate alignment of sequencing reads.

Quantification

HTSeq was used to count the read numbers mapped of each gene, including known and novel genes. And then RPKM of each gene was calculated based on the length of the gene and reads count mapped to this gene. RPKM, Reads Per Kilobase of exon model per Million mapped reads, considers the effect of sequencing depth and gene length for the reads count at the same time, and is currently the most commonly used method for estimating gene expression levels.

Differential Expression Analysis

Differential expression analysis between two conditions/groups (three biological replicates per condition) was performed using DESeq2 R package. DESeq2 provides statistical routines for determining differential expression in digital gene expression data using a model based on the negative binomial distribution. The resulting P values were adjusted using the Benjamini and Hochberg’s approach for controlling the False Discovery Rate (FDR). Genes with an adjusted *p* value < 0.05 found byDESeq2 were assigned as differentially expressed.

Enrichment Analysis

A common way for searching shared functions among genes is to incorporate the biological knowledge provided by biological ontologies. Gene Ontology (GO) annotates genes to biological processes, molecular functions, and cellular components in a directed acyclic graph structure, and Kyoto Encyclopedia of Genes and Genomes (KEGG) annotates genes to pathway.KEGG is a database resource for understanding high-level functions and utilities of the biological system, such as the cell, the organism and the ecosystem, from molecular-level information, especially large-scale molecular datasets generated by genome sequencing and other high-through put experimental technologies (http://www.genome.jp/kegg/). We used KOBAS software to test the statistical enrichment of differential expression genes in KEGG pathways.

**Figure S1.** The selected gRNAs induced mutations in the Vhl locus. After transfection of the mpkCCD cells with the virus particles for gRNA1-3 and the scrambled gRNA, the region covering the location of the gRNA sequences in the Vhl locus was amplified using specific primers. The PCR products were analyzed by Sanger sequencing. The electropherograms of the gRNA1-3 showed compared to Scr multiple peaks in the region of the gRNA sequence (light blue). Dark blue labels the PAM sequence.

**Figure S2.** TIDE analysis leads to identification of clones with frame shift mutations. After Bulk transduction of mpkCCD cells, single cell clones were selected and analyzed for mutations in the Vhl-locus as described above. For positive clones the type of mutation was analyzed using the Tracking of Indels by Decomposition tool ^1^. The results showed that the single cell clone H6 showed a 1bp insertion and a 22 bp deletion. The single cell clone G10 showed a 1 and 4 bp deletion.

**Figure S3.** Effect of Vhl deletion on selected EMT marker genes. The expression of fibronectin (Fn1), Alpha smooth actinin (α-Sma), N-cadherin (N-Cad) and vimentin (Vim) was analyzed in Vhl deficient cells compared to Scr cells by real time PCR. Significant differences were calculated by students t-test and are marked by * (p < 0.5, n = 3).

**Figure S4.** Hyper osmolality increases doubling time. Cells were cultivated in 96 well plates either at 300 mosmol/kg or challenged with hyper osmotic medium and the proliferation was measured by live cell imaging using IncuCyte S3 system. The relative cell density was plotted and the doubling time was calculated by nonlinear exponential growth equation using GraphPad Prism (A). The mean doubling times are plotted. One Way ANOVA was performed to identify statistically significant differences (p value < 0.05; n > 5).

**Figure S5.** VHL expression has no effect on proliferation rate of 786-0 RCC cell line. 786-0 and 786-0-VHL cells were cultivated in 96 well plates and the proliferation was measured by live cell imaging using IncuCyte S3 system. The relative cell density was plotted and the doubling time was calculated by nonlinear exponential growth equation using GraphPad Prism. One Way ANOVA was performed to identify statistically significant differences (p value < 0.05; n > 5).

**Figure S6:** VHL deletion increases migration speed. Scr Cells and Vhl deleted cells (G10 and H6) were cultivated in 96 well plates either and the migration was measured by live cell imaging using IncuCyte S3 system. The upper panel shows the wound at time point 0 h and the lower panel at time point 16 h. Uncovered areas are labeled in green and covered areas in blue.

**Figure S7.** Ectopic VHL expression induces cell migration capacity in 786-0 cells. Cells were cultivated in 96 well plates and a wound to the cell monolayer was applied using the AutoScratch wound making tool. The cell migration was observed by live cell imaging using the IncuCyte S3 system. The relative wound density was plotted and the relative wound density after 12 h was calculated by linear regression analysis using GraphPad Prism. The migration speed was normalized to the Scr cells. One Way ANOVA was performed to identify statistically significant differences (p value < 0.05; n > 3).

**Figure S8.** The expression of Aqp2 mRNA is down regulated in Vhl-KO cells. Scr and Vhl-KO cells (G10, H6) were cultivated at 600 mosmol/kg. Total RNA was extracted, real time PCR performed and relative Aqp2 expression compared to Scr cells was calculated. One Way ANOVA was performed to identify statistically significant differences (*p*-value < 0.05; n > 3).


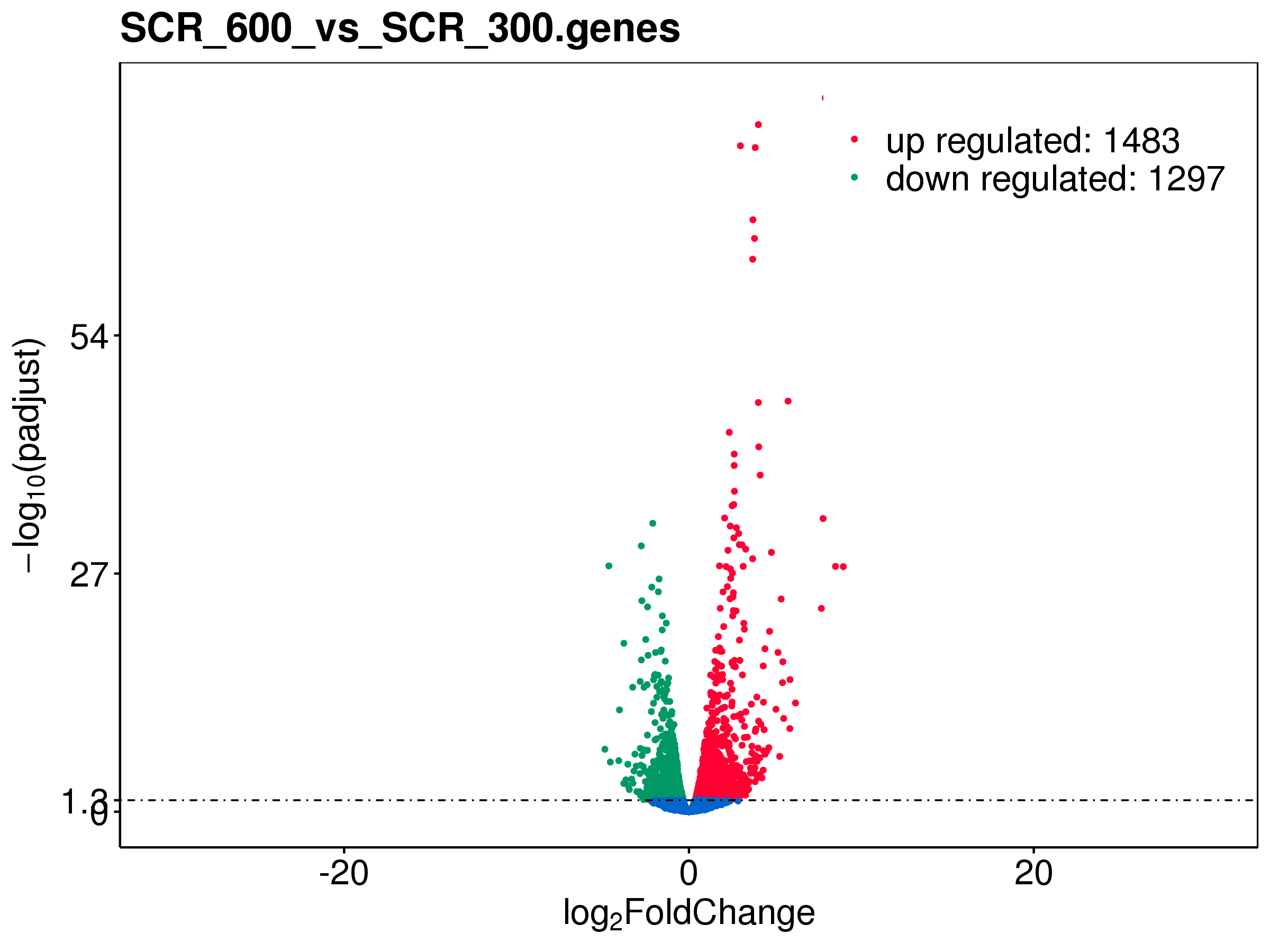


**Figure S9.** Hyper osmolality causes massive changes in gene expression. Scr cells were cultivated at 300 or 600 mosmol/kg. Total RNA was isolated and gene expression was analyzed using Next Generation Sequencing technology and differentially expressed genes were identified (p < 0.005, n=3). The volcano plots show the number of genes, the p-values and log2 fold changes for cells cultivated at 600 vs. 300 mosmol/kg.

**Figure S10.** KEGG pathway analysis. The list of differentially expressed genes between Vhl-KO and Scr cells cultivated under isotonic (300 mosmol/kg) in der upper panel and under hyper osmotic conditions (600 mosmol/kg) in the lower panel were analyzed for enrichment of KEGG pathways. The top 20 enriched pathways are presented here.

**Figure S11.** Kaplan-Meier analysis of survival probability for selected genes. The TCGA KIRC database were queried for selected genes if they have an impact on survival probability using the UCSC Xena Browser (https://xenabrowser.net/heatmap/). The quartiles for expression level were used.

**Figure S12.** Deletion of Vhl induces the expression of hyper osmolality suppressed genes with unfavorable prognostic value. The list of genes that are 1) suppressed by hyper osmolality, 2) unfavorable for patients outcome and 3) differentially expressed in Vhl-KO cell with a log2 fold change of 1 or higher and -1 or are plotted here.

**Figure S13.** Total Western blot membrane from figure 1.

Reference

1. Brinkman, E.K.; Chen, T.; Amendola, M.; van Steensel, B. Easy quantitative assessment of genome editing by sequence trace decomposition. *Nucleic Acids Research* **2014,** *42*, e168-e168.
